# The synergistic effects of a leaf mixture on decomposition change with a period of terrestrial exposure prior to immersion in a stream

**DOI:** 10.1002/ece3.10959

**Published:** 2024-03-05

**Authors:** Manuela Abelho, Enrique Descals

**Affiliations:** ^1^ Polytechnic Institute of Coimbra Coimbra Agriculture School Coimbra Portugal; ^2^ Associate Laboratory TERRA, Calçada Martim de Freitas Centre for Functional Ecology – Science for People and the Planet Coimbra Portugal; ^3^ Instituto Mediterráneo de Estudios Avanzados, IMEDEA (CSIC) Esporles Spain

**Keywords:** cross‐ecosystem flows of organic matter, decomposers, detritivores, fungi, mass loss, nonadditive effects, shredders, stream, terrestrial‐aquatic exposure

## Abstract

The effect of mixing litter on decomposition has received considerable attention in terrestrial and aquatic (but rarely in both) ecosystems, with a striking lack of consensus in the obtained results. We studied the decomposition of a mixture of poplar and alder in three terrestrial: aquatic exposures to determine (1) if the effect of mixing litter on mass loss, associated decomposers (fungal biomass, sporulation rates, and richness), and detritivores (abundance, biomass, and richness of invertebrate shredders) differs between the stream (fully aquatic exposure) and when litter is exposed to a period of terrestrial exposure prior to immersion and (2) the effect of the mixture across exposure scenarios. The effect of the mixture was additive on mass loss and synergistic on decomposers and detritivores across exposure scenarios. Within scenarios, mass loss and decomposers showed synergistic effects only in the fully aquatic exposure, detritivores showed synergistic effects only when the period of terrestrial was shorter than the period of aquatic exposure, and when the period of terrestrial was equal to the period of aquatic exposure the effect of the mixture was additive on mass loss, decomposers, and detritivores. The species‐specific effects also differed among exposure scenarios. Alder affected poplar only when there was a period of terrestrial exposure, with increased sporulation rates and fungal richness in exposure 25:75, and increased mass loss in exposure 50:50. Poplar affected alder only under fully aquatic exposure, with increased mass loss. In conclusion, the synergistic effects of the mixture changed with a period of terrestrial exposure prior to immersion. These results provide a cross‐boundary perspective on the effect of mixing litter, showing a legacy effect of exposure to terrestrial decomposition on the fate of plant litter in aquatic ecosystems and highlighting the importance of also assessing the effect of mixing litter on the associated biota and not only on mass loss.

## Graphical abstract

The effect of mixing litter on stream decomposition changed with a period of terrestrial exposure before immersion. The mixture had synergistic effects on mass loss and decomposers under fully aquatic exposure, synergistic effects on detritivores when the period of terrestrial was shorter than the period of aquatic exposure, and when the period of terrestrial and aquatic exposures were equal, the effect of the mixture was additive.

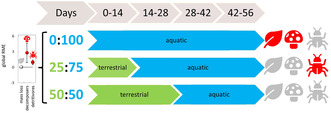

## 1 INTRODUCTION

Leaf litter from forests supports complex detritus‐based food webs in forest floors and streams through decomposition by the activity of microbial decomposers and invertebrate detritivores (Gessner et al., 2010). While the decomposition process has been mostly studied on monocultures, Seastedt (1984) suggested that litter mixtures might decompose at different rates from those predicted from single species (i.e., showing nonadditive effects) due to the effect of resource heterogeneity on microbial decomposers and invertebrate detritivores. This catapulted research on the effect of mixing litter. Nonadditive effects may be due to leaf litter chemical characteristics, such as compounds which translocate from one leaf species and may enhance (e.g., nitrogen) or inhibit (e.g., polyphenols) decomposition of other species (Lummer et al., 2012; Sanpera‐Calbet et al., 2009; Schimel & Hättenschwiler, 2007). Nonadditive effects may also be due to leaf litter physical characteristics, such as higher leaf toughness of one species, which may act as an armoring effect against physical abrasion, thus decreasing decomposition of other more fragile species (Swan et al., 2008; Wardle et al., 1997), and synergistic effects may also arise from the higher habitat/resource heterogeneity of mixtures when compared to single species (Epps et al., 2007), which enhances colonization by biota and eventually results in higher overall processing rates by leaf consumers (Bastian et al., 2008).

The effect of mixing leaf species may reveal important consequences of tree biodiversity loss for the functioning and biodiversity of the recipient ecosystem (e.g., Gessner et al., 2010), but the subject is interesting per se, as similar studies, either in terrestrial, aquatic, or both ecosystems, often present strikingly contrasting results. This is well illustrated by five recent meta‐analyses. Kou et al. (2020), using a data set from 65 studies in forest ecosystems across biomes, concluded that N release (but not P) and mass loss were higher when litter was composed of two to three species. In contrast, Porre et al. (2020) found, in 78 studies across terrestrial ecosystems worldwide, that most litter mixtures had close to additive mass loss and only 15% of the data showed a significant nonadditive mass loss in mixture, thus concluding that interactive effects among litter species are contextual and cannot be generalized beyond the context in which the results are obtained. Similarly, Chen and Chen (2021) found that the C:N:P ratios of plants, soil organic matter, microbial biomass, and enzymes did not differ significantly between mixtures and monocultures in 169 studies in terrestrial ecosystems. Across biomes and ecosystems, Liu et al. (2020) compared 69 studies and concluded that the effect of mixing litter demonstrates nonadditive effects on decomposition rates in terrestrial and aquatic ecosystems, and that those effects are most frequently synergistic. Finally, Mori et al. (2020), using a data set from 151 studies across biomes and ecosystems, found synergistic litter species‐mixing effects on decomposition rates for most ecosystems, including aquatic, but no effect in streams. Thus, the existence of a general, nonadditive relationship between litter mixing and the process of decomposition, both within and across terrestrial and aquatic ecosystems, remains elusive.

The flow of organic matter from the terrestrial to the aquatic habitats is the basis of energy and matter for many stream ecosystems (Marks, 2019; Wallace et al., 1997), with leaf litter falling vertically or entering the water by lateral movement through the forest floor (Abelho, 2001; Benfield, 1997). Abelho and Descals (2019) assessed leaf decomposition of two species of contrasting chemistry common in the study area, alder (*Alnus glutinosa* (L.) Gaertn.), hybrid black poplar (*Populus × canadensis* Moench), and a 1:1 mixture of the two species in three scenarios across a gradient of terrestrial: aquatic exposures, to determine if decomposition of leaf litter from lateral litter inputs to streams was similar to that of vertical inputs and concluded that decomposition rates and microbial and invertebrate colonization decreased with the increase in the period of terrestrial exposure for all litter types. Does the effect of mixing litter on decomposition also differ between lateral and vertical inputs? Using the data set from Abelho and Descals (2019), the objective of this work was to assess (1) if the effect of mixing leaf litter on the process of decomposition (mass loss, associated decomposers, and detritivores) differs between the stream ecosystem (under fully aquatic exposure) and when litter is exposed to a period of terrestrial exposure prior to immersion in the stream and (2) the effect of the mixture across exposure scenarios. Based on the meta‐analyses cited above, the tested hypotheses were: (a) the effect of the mixture is additive under fully aquatic exposure, given the results of Mori et al. (2020) for streams; (b) the effect of the mixture is synergistic for leaf litter with a period of terrestrial exposure, given the predominance of synergistic effects found by Liu et al. (2020) across ecosystems; and (c) synergistic effects are due to the enhancement of the decomposition process of poplar by the presence of alder, given the stimulating effects of N‐fixing species on the decomposition of N‐poor species (Hättenschwiler & Gasser, 2005).

## 2 MATERIALS AND METHODS

### 2.1 Site, experimental design, methods, and data

The experiment was carried out at Ribeira do Botão, a 3rd‐order stream located near Coimbra, Central Portugal (40°18′23″ N, 08°23′55″ W; elevation 80 m) during 56 days from November to December 2011. The riparian vegetation was composed of mixed deciduous trees, mainly common alder. During the exposure period (data collected at a nearby meteorological station located at 40°12′33″ N; 08°27′08″ W; elevation 16 m), daily air temperature ranged 4.8–19.9°C, humidity ranged 54%–96%, and precipitation totaled 241 mm. In the stream, water was well‐oxygenated (11.9–13.0 mg L^−1^), temperature ranged 5.9–13.4°C, pH was circumneutral (6.6–7.3), nitrate ranged 0.45–2.55 mg NO_3_^−^‐N L^−1^, ammonia ranged 21.5–94.8 μg NH_4_^+^‐N L^−1^, phosphate ranged 79.7–109.4 μg PO_4_^3−^‐P L^−1^, and current velocity ranged 0.239–0.458 m s^−1^.

**FIGURE 1:**
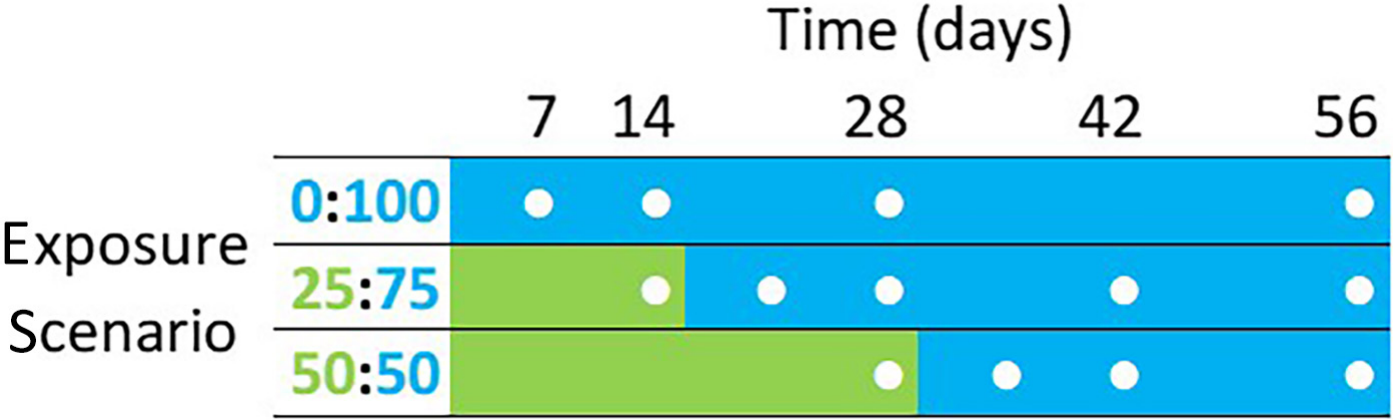
Experimental design and sampling scheme (white dots, three replicates) of the litterbags in the three exposure scenarios: 0:100 (0 days terrestrial followed by 56 days aquatic), 25:75 (14 days terrestrial followed by 42 days aquatic), and 50:50 (28 days terrestrial followed by 28 days aquatic). The day 0 samples were used to assess initial oven‐dry mass of the other samples. On the transition between the terrestrial and the aquatic environment, one set of samples was collected for assessing decomposition on the terrestrial environment.

Other characteristics of the study site and the litter decomposition study are described in detail in Abelho and Descals (2019). Briefly, decomposition of senescent leaf litter (2.80 g ± 0.52 SD) of the N‐fixing alder, hybrid black poplar, and a 1:1 mixture of both species was assessed on litterbags (5 mm mesh) exposed to both the terrestrial and the aquatic environment according to three exposure scenarios (Figure 1): 0:100 (0 days terrestrial followed by 56 days aquatic), 25:75 (14 days terrestrial followed by 42 days aquatic), and 50:50 (28 days terrestrial followed by 28 days aquatic). The riparian litterbags were deployed on the floor next to the stream channel (approximately 1.5 m above the stream surface and 1 m away from the stream margin), and loosely covered with dead fallen leaves. The stream bags were tied to iron nails fixed at the bottom in a riffle area. Three additional similar portions of each litter type were used to determine the initial oven‐dry mass (60°C, 3 days) of the leaves used in the experiment. Sampling in stream (0:100) was carried out after 7, 14, 28, and 56 days of immersion. Sampling in the terrestrial environment occurred after 14 (25:75) and 28 days (50:50) of exposure; three replicates of each litter type were used to determine litter dry mass remaining and litter colonization in the terrestrial environment, and the other groups were transferred to the riffle area of the stream and sampled after 7, 14, 28 and 42 days (25:75) or after 7, 14, and 28 days of immersion (50:50).

On each sampling occasion, mass loss of leaf litter (% dry mass), ergosterol as an estimate of fungal biomass (mg g_AFDM_^−1^), aquatic hyphomycete sporulation rates (number of conidia mg_AFDM_^−1^ day^−1^) and taxa richness (number of species sample^−1^), shredder biomass (mg bag^−1^), abundance (number of individuals bag^−1^), and taxa richness (number of taxa bag^−1^) were determined on three replicates as described in Abelho and Descals (2019). In the mixture, mass loss and the fungal variables were determined for each species; to obtain the value in the mixture, mass loss was summed, and the fungal variables were averaged.

The two leaf species differed significantly in their initial chemical characteristics, decomposition rates, and colonization patterns by decomposers and detritivores (Table 1), as determined by Abelho and Descals (2019).

**TABLE 1:** Initial leaf litter chemistry (mean ± 1 SE), decomposition rates (± 95% CL), fungal and shredder colonization (mean ± 1 SE) of alder, the mixture, and poplar in the three exposure scenarios (data from Abelho & Descals, 2019).

	Alder	1:1 mixture	Poplar
Leaf litter chemistry			
%N	2.33 ± 0.026	1.53 ± 0.009	0.73 ± 0.009
C:N	23.46 ± 0.284	45.53 ± 0.564	67.59 ± 1.389
Decomposition rates (−*k* day^−1^)			
0:100	0.0566 ± 0.0166	0.0333 ± 0.0046	0.0234 ± 0.0042
25:75	0.0418 ± 0.0063	0.0243 ± 0.0041	0.0145 ± 0.0010
50:50	0.0381 ± 0.0114	0.0198 ± 0.0040	0.0129 ± 0.0016
Fungal biomass (mg_ergosterol_ g_AFDM_^−1^)			
0:100	2.82 ± 0.89	2.15 ± 0.30	3.69 ± 0.86
25:75	1.00 ± 0.57	1.06 ± 0.42	3.36 ± 1.52
50:50	5.12 ± 2.92	0.83 ± 0.18	5.05 ± 4.29
Fungal sporulation rates (no. conidia mg_AFDM_^−1^ day^−1^)			
0:100	534.7 ± 250.1	90.0 ± 40.5	61.4 ± 32.1
25:75	1.68 ± 0.09	8.88 ± 0.43	8.63 ± 0.30
50:50	1.28 ± 0.38	2.90 ± 1.01	0.51 ± 0.22
Fungal richness (no. taxa bag^−1^)			
0:100	7.42 ± 0.49	10.43 ± 0.90	7.44 ± 1.43
25:75	3.00 ± 0.71	6.50 ± 0.42	3.91 ± 0.94
50:50	2.00 ± 1.00	3.00 ± 1.15	1.78 ± 0.62
Shredder biomass (mg bag^−1^)			
0:100	7.32 ± 2.06	9.23 ± 2.60	6.38 ± 1.01
25:75	5.22 ± 1.34	10.57 ± 1.96	5.77 ± 1.48
50:50	7.16 ± 2.17	7.42 ± 2.34	12.13 ± 5.44
Shredder abundance (no. bag^−1^)			
0:100	36.92 ± 9.66	37.25 ± 9.95	30.25 ± 3.28
25:75	17.80 ± 5.17	25.13 ± 5.84	22.00 ± 5.79
50:50	24.92 ± 7.34	24.08 ± 5.78	18.33 ± 4.03
Shredder richness (no. taxa bag^−1^)			
0:100	2.00 ± 0.25	2.00 ± 0.25	1.83 ± 0.21
25:75	2.80 ± 0.44	3.40 ± 0.35	2.73 ± 0.37
50:50	3.08 ± 0.36	3.00 ± 0.46	3.00 ± 0.43

### 2.2 Calculations and statistical analysis

The effect of mixing litter on each response variable was assessed by computing, for each pair of replicates, the deviation between observed (*O*) and expected (*E*) values: (*O*−*E*)/*E*. The relative mixture effect (RME; Wardle et al., 1997) was calculated as the deviation between the values in the mixture (*O*) and the average values of the two single species (*E*). To detect any species‐specific effects of the mixture, the relative individual performance (RIP; Zhou et al., 2020) was calculated as the deviation between the values of mass loss and fungal variables of a species in the mixture (*O*) and the values of the same single species (*E*). RME and RIP were considered significantly different from zero (i.e., nonadditive) when the mean was bigger than its 95% confidence limits (CL), that is, when the 95% CL did not overlap 0 in the graphs, synergistic if the deviation was positive or antagonistic if the deviation was negative (Ball et al., 2008).

To provide a measure of the effect of the mixture on colonization by each of the biota associated with leaf litter, an “RME/RIP decomposers” and an “RME detritivores” were calculated as above, with the same weight for each of the three fungal variables and of the three shredder variables, respectively. The effect of the mixture across exposure scenarios was assessed with a “global RME/RIP,” calculated with the same weight for each of the three exposures to overcome the different number of observations among exposure scenarios. To capture the effect of mixing litter on the process of decomposition, defined as “all biological processes contributing to organic matter mass loss and transformation” (Gessner et al., 2010), an integrated measure containing the effects on mass loss and associated biota – “RME/RIP processing” – was calculated as above with the same weight for mass loss, decomposers, and detritivores.

To examine all sources of variation of the values of the response variables, a mixed model analysis of variance (GLM ANOVA) was used to assess the effect of: (1) mixture [are observed values similar to expected ones?]; (2) exposure scenario [are observed and expected values similar among the three exposure scenarios?]; and (3) interaction 1 × 2 [does the effect of mixture depend on exposure scenario?]. Due to the different number of sampling occasions among exposures, ANOVA was calculated with type III SS (Shaw & Mitchell‐Olds, 1993). In case of a significant interaction, one‐way ANOVA was carried out as above to detect the effect of the levels of each factor. Because the identity of the sampling days varied among exposures, time was set as a random factor. Tukey's HSD test was used for pairwise comparisons after a significant exposure scenario effect (Zar, 2010). All data were tested for parametrical assumptions; heteroskedastic variables (Levene's test) were transformed with the square root (counts) or with the natural logarithm (all other variables; Zar, 2010). The statistical analyses were performed with the software STATISTICA 13.0 with the level of significance set at *p* = .05.

## 3 RESULTS

### 3.1 Global effect of the mixture across exposure scenarios

**FIGURE 2:**
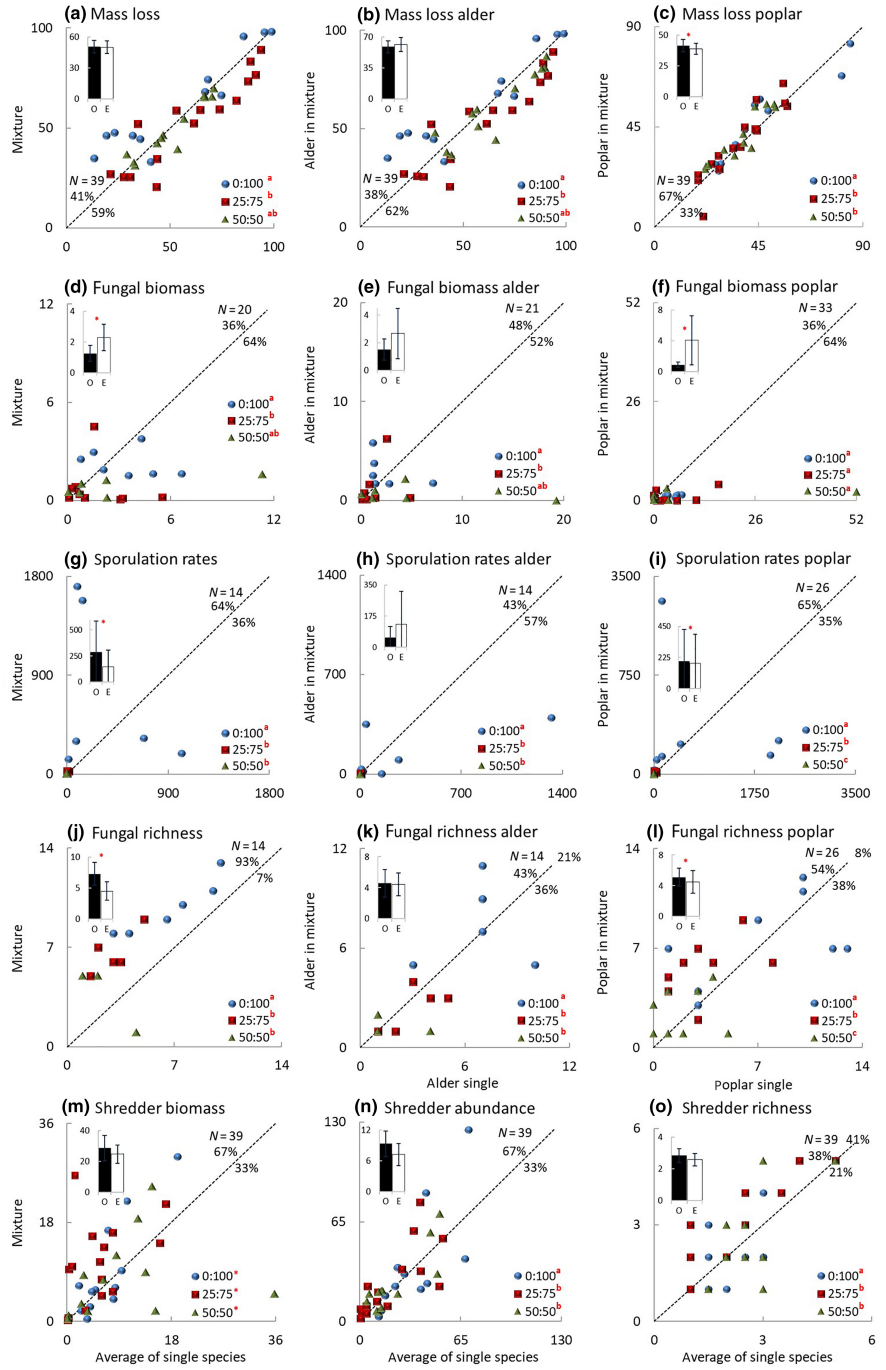
Relationship between expected and observed values during 56 days decomposition under the three terrestrial:aquatic exposure scenarios: 0:100 (100% aquatic), 25:75 (25% terrestrial and 75% aquatic), and 50:50 (50% terrestrial and 50% aquatic). (a–c) % mass loss; (d–f) mg ergosterol g_AFDM_^−1^; (g–i) number of conidia g_AFDM_^−1^ day^−1^; (j, k, l, n, o) number bag^−1^; (m) mg bag^−1^. The dashed line represents 1:1 (no difference between expected and observed values). *N* = number of data‐pairs and percentage of values higher‐than‐expected (above the 1:1 line), lower‐than‐expected (below the 1:1 line), or equal‐to‐expected (on the 1:1 line). *Inset* shows average (±95% CL) of observed (*O*) and expected (*E*) values across the three exposure scenarios, an asterisk indicating significant differences after two‐way ANOVA (Table 2). Exposure scenarios with different superscript letters are significantly different after Tukey HSD test; an asterisk indicates a significant effect not detected by Tukey's HSD test.

**FIGURE 3:**
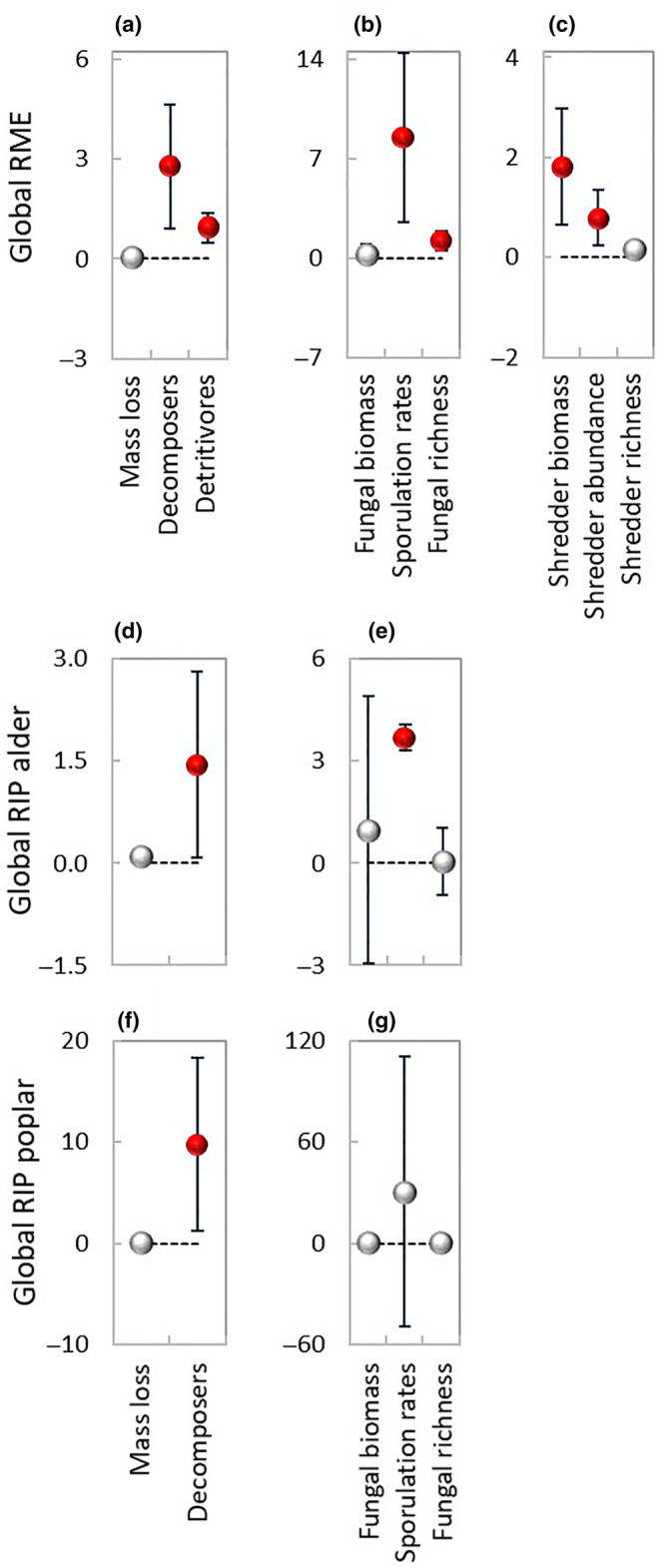
Global relative mixture effect (RME, mean ± 95% CL) and relative individual performance (RIP, mean ± 95% CL) on the study variables during 56 days decomposition under the three terrestrial:aquatic exposure scenarios, 0:100 (100% aquatic), 25:75 (25% terrestrial and 75% aquatic), and 50:50 (50% terrestrial and 50% aquatic). Closed red symbols denote significant RME or RIP effects, that is, the 95% CL do not cross the zero line. Number of data‐pairs: (a) 39, 50, 117; (b) 22, 14, 14; (c) 39, 39, 39; (d) 39, 49; (e) 21, 14, 14; (f) 39, 85; (g) 33, 26, 26.

There were no significant differences between expected and observed values of mass loss (Figure 2a), and the relative mixture effect was additive across exposure scenarios (Figure 3a). Most data points of alder distributed below (Figure 2b) while most data points of poplar distributed above the 1:1 line (Figure 2c), resulting in a significant interaction between mixture effect and exposure scenario (Table 2). Despite a significant difference between mass loss of poplar in the mixture and as single species (*F*_1,66_ = 5.17, *p* < .05; Table 2), the relative individual performance was additive for both species (Figure 3d,f).

**TABLE 2:** Summary of the ANOVA tables (GLM Type III SS; time as a random factor) for the effects of mixture, exposure scenario, and their interaction on the decomposition variables, after transformation of heteroskedastic data as shown.

Source of variation	df	Mixture effect		Exposure effect		Tukey	Interaction	
		*F*	*p*	*F*	*p*		*F*	*p*
RME								
Mass loss	66	0.04	.84*	63.20	**<.00001** *	0:100 > 25:75 = 50:50	3.85	**<.05**
ln(fungal biomass)	34	5.26	**<.05** (*O* < *E*)	7.12	**<.01**	0:100 > 25:75 = 50:50	0.22	.81
ln(fungal sporulation rate)	19	6.75	**<.05** (*O* > *E*)	12.72	**<.01**	0:100 > (25:75 = 50:50)	0.16	.85
Fungal taxa richness	19	18.67	**<.001** (*O* > *E*)	11.44	**<.01**	0:100 > (25:75 = 50:50)	1.23	.31
ln (shredder biomass)	66	1.31	.26	3.61	**<.05**	Not detected	1.75	.18
SQR (shredder abundance)	66	0.92	.34	9.15	**<.001**	0:100 > (50:50 = 25:75)	0.39	.68
SQR (shredder taxa richness)	66	0.80	.38	18.92	**<.00001**	0:100 < (50:50 = 25:75)	1.11	.34
RIP alder								
Mass loss	65	0.41	.52*	14.81	**<.00001** *	50:50 > 25:75 = 0:100	5.24	**<.01**
ln (fungal biomass)	30	0.09	.77	5.90	**<.01**	0:100 > 25:75 = 50:50	0.56	.58
ln (fungal sporulation rate)	21	0.08	.78	8.03	**<.01**	0:100 > (25:75 = 50:50)	0.11	.89
SQR (fungal taxa richness)	21	0.13	.72	19.72	**<.001**	0:100 > (25:75 = 50:50)	0.67	.52
RIP poplar								
Mass loss of poplar	64	5.17	**<.05** (*O* > *E*)	111.83	**<.00001**	0:100 > (50:50 = 25:75)	0.28	.76
ln (fungal biomass)	52	7.36	**<.01** (*O* < *E*)	1.41	.25	–	0.81	.45
ln (fungal sporulation rate)	40	5.91	**<.05** (*O* > *E*)	17.09	**<.00001**	0:100 > 50:50 > 25:75	0.02	.99
SQR (fungal taxa richness)	40	4.75	**<.05** (*O* > *E*)	6.96	**<.01**	0:100 > 50:50 > 25:75	0.98	.38

*Note*: RME, relative mixture effect, where observed are the values in the mixture and expected are the average values of the single species; RIP, relative individual performance, where observed are the values for the species in the mixture and expected are the values for that single species; df, degrees of freedom of the denominator; ln, natural logarithm transformation (*x*′ = ln (*x* + 1)); SQR, square root transformation (*x*′ = √(*x* + ^3^/_8_)). Significant effects are shown in bold.

* Results of one‐way ANOVA after a significant interaction between mixture and exposure effects.

Sporulation rates and fungal richness were significantly higher (*F*_1,19_ > 6.75, *p* < .05; Figure 2g,j; Table 2), while fungal biomass was significantly lower (*F*_1,34_ = 5.26, *p* < .05; Table 2, Figure 2d) in the mixture than the average of the single species. Overall, the relative mixture effect on decomposers was synergistic (RME: 0.91–4.64) across exposure scenarios (Figure 3a), due to increased sporulation rates (RME: 2.51–14.44) and richness (RME: 0.56–1.90) while the effect on biomass was additive (Figure 3b). For alder, there were no significant differences between expected and observed values of any of the fungal variables (Figure 2e,h,k). For poplar, sporulation rates and richness were significantly higher (*F*_1,40_ > 4.75, *p* < .05; Table 2, Figure 2i,l) while fungal biomass was significantly lower (*F*_1,52_ = 7.36, *p* < .01; Table 2, Figure 2f) in the mixture than as single species. When all three fungal variables were considered together, the relative individual performance on decomposers was synergistic for both species (Figure 3d,f), weaker for alder (RIP: 0.09–2.81), and stronger for poplar (RIP: 1.17–18.23). However, the effect on each of the fungal variables differed between the two species. In the case of alder, there was a huge variation in the deviation between observed and expected values of fungal biomass, with a synergistic effect for sporulation rates only (Figure 3e), while in the case of poplar there was a huge variation in the deviation between observed and expected values of sporulation rates with additive effects for all three fungal variables (Figure 3g).

Most data points of shredder colonization were distributed above the 1:1 line (Figure 2m,n,o) resulting in a synergistic relative mixture effect (RME: 0.48–1.37) on detritivores across exposure scenarios (Figure 3a), due to increased shredder biomass (RME: 0.66–2.98) and abundance (RME: 0.23–1.35) while the effect on richness was additive (Figure 3c).

The relative mixture effect on the process of decomposition (Figure 6) was globally synergistic across exposure scenarios (RME: 0.70–1.79) due to the increased decomposer and detritivore colonization.

### 3.2 Effect of the mixture within exposure scenarios

**FIGURE 4:**
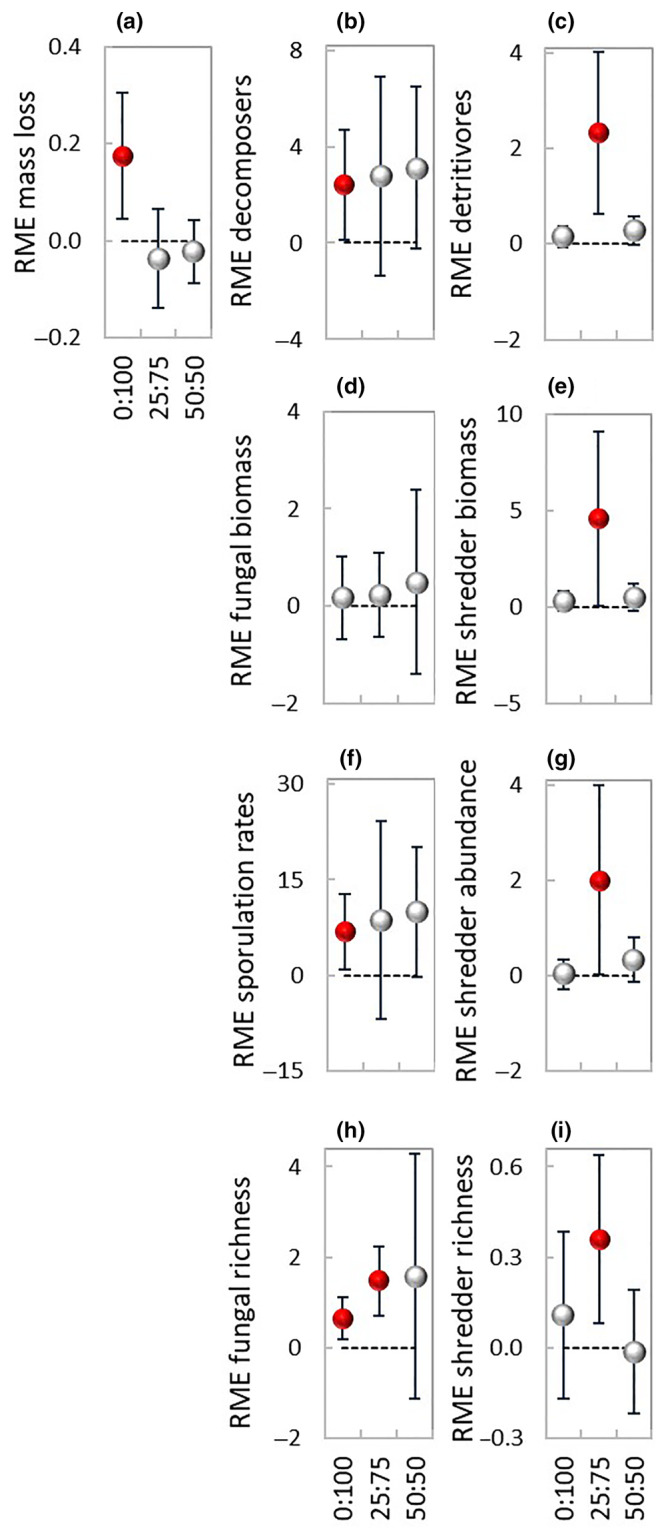
Relative mixture effect (RME, mean ± 95% CL) on the study variables during 56 days decomposition under each of the three terrestrial:aquatic exposure scenarios, 0:100 (100% aquatic), 25:75 (25% terrestrial and 75% aquatic), and 50:50 (50% terrestrial and 50% aquatic). Closed red symbols denote significant RME effects, that is, the 95% CL do not cross the zero line. Number of data‐pairs: (a, e, g, i) 12, 15, 12; (b) 19, 19, 12; (c) 36, 45, 36; (d) 7, 9, 6; (f, h) 6, 5, 3.

**FIGURE 5:**
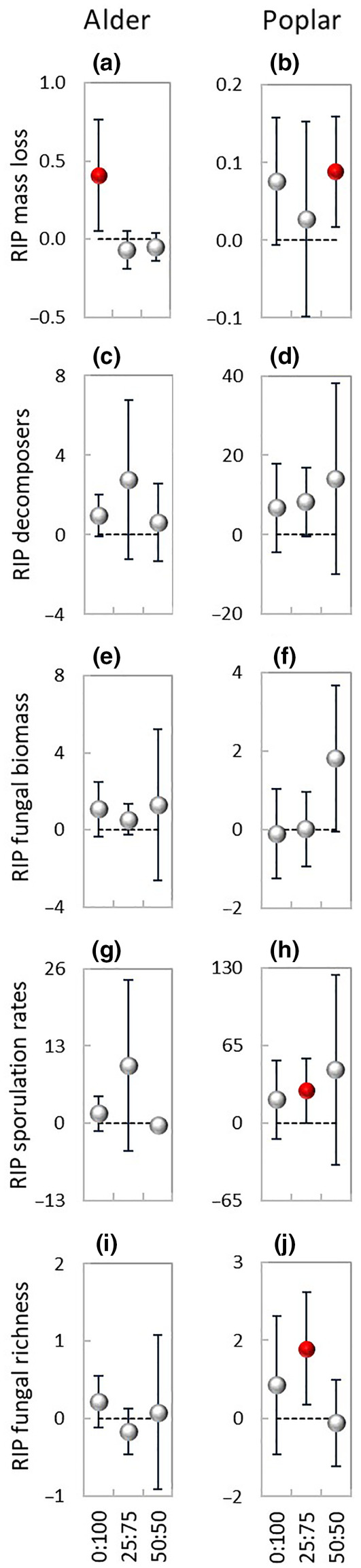
Relative individual performance (RIP, mean ± 95% CL) on the study variables for alder and poplar leaf litter during 56 days decomposition under each of the three terrestrial:aquatic exposure scenarios, 0:100 (100% aquatic), 25:75 (25% terrestrial and 75% aquatic), and 50:50 (50% terrestrial and 50% aquatic). Closed red symbols denote significant RIP effects, that is, the 95% CL do not cross the zero line. Number of data‐pairs: (a) 12, 15, 12; (b, c) 18, 19, 12; (d) 27, 28, 30; (e) 6, 9, 6; (f) 9, 12, 12; (g, j) 6, 5, 3; (h, i) 9, 8, 9.

Most mass loss data points of exposure 0:100 distributed above while most data points of exposures 25:75 and 50:50 distributed below the 1:1 line (Figure 2a), resulting in a significant interaction between mixture effect and exposure scenario (Table 2) and in a weak synergistic relative mixture effect (RME: 0.04–0.30) in 0:100 exposure only (Figure 4a). The relative individual performance differed between the two species. In the case of alder, most data points of exposure 0:100 distributed above the 1:1 line (Figure 2b); there was an interaction between mixture effect and exposure scenario (Table 2) and a synergistic effect (RIP: 0.05–0.77) occurring in exposure 0:100 only (Figure 5a). In the case of poplar, most data points of exposure 50:50 distributed above the 1:1 line (Figure 2c) with a weak synergistic effect (RIP: 0.02–0.16) occurring in exposure 50:50 only (Figure 5b).

The relative mixture effect on decomposers was synergistic in exposure 0:100 (RME: 0.14–4.69) while in exposures 25:75 and 50:50 the effect was additive (Figure 4b). The synergistic effect found in exposure 0:100 was due to enhanced sporulation rates (RME: 0.81–12.82; Figure 4f) and fungal richness (RME: 0.18–1.11; Figure 4h), while the effect on fungal biomass was additive (Figure 4d). In exposure 25:75, there was a synergistic relative mixture effect on fungal richness (RME: 0.70–2.23). When all fungal variables were taken together, the effect of the mixture on decomposers was additive for both species in all exposure scenarios (Figure 5c,d). However, while for alder the effect was additive for all fungal variables (Figure 5e,g,i), for poplar the effect was additive for fungal biomass (Figure 5f), but there was a strong synergistic effect on sporulation rates (RIP: 0.37–54.47) and a weaker one on richness (RIP: 0.26–2.42) in exposure 25:75 (Figure 5h,j).

The effect of the mixture on detritivores (Figure 4c) was additive in exposures 0:100 and 50:50 and synergistic in exposure 25:75 (RME: 0.63–4.02), where all three shredder variables had higher‐than‐expected values (Figure 4e,g,i), with effects stronger for biomass (RME: 0.09–9.14) and abundance (RME: 0.01–3.99) than for richness (RME: 0.08–0.64).

**FIGURE 6:**
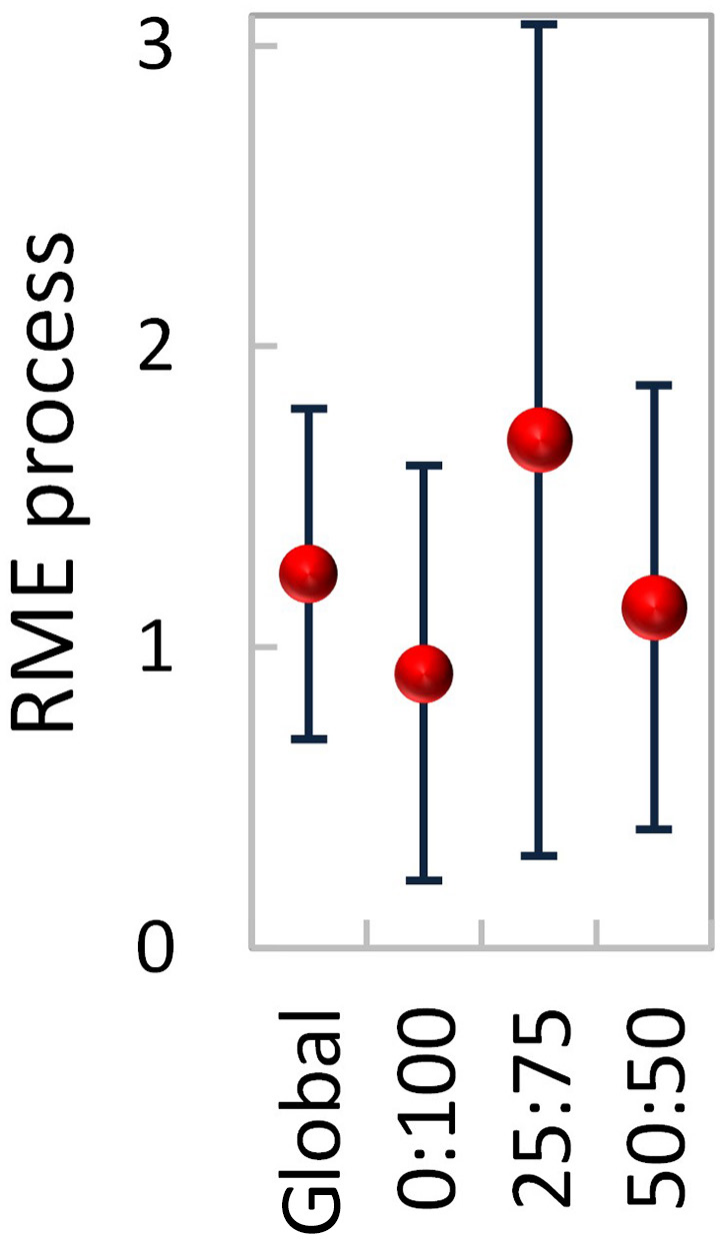
Relative mixture effect (RME, mean ± 95% CL) on the process of decomposition (1/3 mass loss, 1/3 decomposers, and 1/3 detritivores) during 56 days, globally across scenarios (global) and under each of the three terrestrial:aquatic exposure scenarios, 0:100 (100% aquatic), 25:75 (25% terrestrial and 75% aquatic), and 50:50 (50% terrestrial and 50% aquatic). Closed red symbols denote significant RME effects, that is, the 95% CL do not cross the zero line. Number of data‐pairs: 206, 67, 79, 60.

The relative mixture effect on the process of decomposition (mass loss, decomposers, and detritivores) was synergistic within all exposure scenarios (Figure 6), with effects stronger in exposures 25:75 (RME: 0.31–3.07) and 50:50 (RME: 0.39–1.87) than in exposure 0:100 (RME: 0.22–1.60).

## 4 DISCUSSION

The obtained results provide a cross‐boundary perspective on the effect of mixing litter, showing a legacy effect of exposure to terrestrial decomposition on the fate of plant litter in aquatic ecosystems, and highlighting the importance of assessing the effect on microbial decomposers and detritivores and not only on mass loss. The hypothesis that the effect of the mixture is additive under fully aquatic exposure was rejected, as an additive effect occurred only on detritivores while the effect on mass loss and on decomposers was synergistic. The hypothesis that the effect of the mixture is synergistic when exposed in the terrestrial and the aquatic habitats was only partially accepted, as the synergistic effect occurred only on decomposers and detritivores while the effect on mass loss was additive. The hypothesis that the synergistic effects are due to an enhancement of the decomposition process of poplar by the presence of alder was only partially accepted. The presence of alder affected poplar only when there was a period of terrestrial exposure, with increased sporulation rates and fungal richness in exposure 25:75, and increased mass loss in exposure 50:50. The presence of poplar affected alder only under fully aquatic exposure, with increased mass loss of alder. Partitioning the effects on both species in the mixture allowed distinguishing if additive effects were due to no change on mass loss and fungi of both species or to opposite nonadditive effects of each species (Hui & Jackson, 2009). Without both assessments – on the mixture and on its component species – nonadditive effects may be masked and interpreted as additive. This could partially explain the apparent contrasting results of Mori et al. (2020) and Liu et al. (2020) across terrestrial and aquatic ecosystems, where the latter also assessed the species‐specific effects of the mixture and found a predominance of synergistic effects on all ecosystems while the former found the effect to be additive in streams.

The main objective of this study was to assess if a period of terrestrial exposure prior to immersion in the stream altered the effect of mixing litter when compared to fully aquatic exposure. While the results, obtained for one mixture at one site, may not extrapolate a trend on a broader context, the mixture used here was the best combination to assess possible nonadditive effects. The number of species in a mixture may not be a good predictor of litter‐quality‐dependent processes and the inclusion of another species may even contribute to the homogenization of the substrate (Epps et al., 2007). Moreover, functional diversity has been considered a better predictor than species diversity (e.g., Bonanomi et al., 2014; Grossman et al., 2020), and highest functional diversity has been found in mixtures of species with contrasting N‐concentrations and C:N ratio (Santonja et al., 2020), which also more frequently show nonadditive synergistic interactions (Bonanomi et al., 2014).

### 4.1 The effect of the mixture is synergistic across exposure scenarios

Synergistic effects dominated the process of decomposition across exposure scenarios, with the number of nonadditive effects largely surpassing the probability of retrieving significant differences by chance. Of the 206 differences between data pairs of expected and observed values (seven litter processing variables across the three exposure scenarios), 92% differed from zero, of which 55% were positive and 37% were negative. The mixture promoted higher‐than‐expected colonization by decomposers (60% of data pairs) and detritivores (57% of data pairs), while mass loss was globally additive (41% of data pairs positive and 59% negative). The effect of the mixture on decomposers resulted mostly from the increased sporulation rates, but, to a lower extent, also from the increased number of species. Concerns on the consequences of biodiversity loss for ecosystem structure and function have driven much of the research on the effect of mixing litter, but interestingly only a few studies include measures of how riparian biodiversity loss may affect diversity of the detritus‐based food webs (but see Abelho, 2009; two studies reviewed by Gartner and Cardon (2004), Chapman et al. (2013)). The synergistic effect of the mixture on fungal richness may indicate that the simplification of riparian diversity will be followed by microbial diversity loss, as suggested by Chapman et al. (2013).

The synergistic effect across exposure scenarios clearly shows that mixing litter stimulated colonization by decomposers and detritivores. However, the effect on mass loss was exposure‐dependent and differed among scenarios, once again highlighting that mass loss or breakdown rates do not fully capture the effect of mixing litter on the process of decomposition.

### 4.2 The effect of the mixture differs among exposure scenarios

Under the fully aquatic exposure, the mixture had a synergistic effect on mass loss and decomposers (sporulation rates and fungal richness), while the effect on detritivores was additive. The synergistic effect on mass loss of the mixture was due to a species‐specific effect on mass loss of alder in the presence of poplar (16% more, on average). Fungi (abundance, activity and/or diversity) may demonstrate evidence for interactive effects of mixtures in streams (López‐Rojo et al., 2020; Santonja et al., 2020; but see Sanpera‐Calbet et al. (2009) and Abril et al. (2021) for contrasting results). Fungi colonizing the mixture were more diverse, probably due to a better substrate for conidia attachment and germination (Dang et al., 2007; Kearns & Bärlocher, 2008) and invested more in reproduction than in biomass when compared to the average of the single species. Aquatic hyphomycetes invest on average 50% of their biomass in sporulation, and the conidia produced may correspond to 10% of the daily litter mass loss (Gessner, 1997). Thus, the increased sporulation rates of the mixture and possibly also positive complementarity among fungal species may explain the increased mass loss (López‐Rojo et al., 2020). Additionally, shredders prefer high‐quality in detriment of poor‐quality leaves (Swan & Palmer, 2006) and may strongly increase the decomposition rates of alder (Bruder et al., 2011). Thus, their feeding activity probably also contributed to the increased mass loss of alder in the presence of poplar and to the synergistic effect of the mixture.

When the period of terrestrial exposure was shorter than the period of aquatic exposure, the mixture had a synergistic effect on detritivores, stronger for shredder biomass and abundance, but not on mass loss or decomposers, although fungal richness was also higher‐than‐expected. On average, the mixture had 63% more fungal species than the average of the monocultures, but the increased richness did not translate into higher mass loss, possibly due to functional redundancy among aquatic hyphomycete species (Bärlocher, 2005; López‐Rojo et al., 2020). On the other hand, invertebrate detritivores have been shown to play a fundamental role on the synergistic effects of mixtures on mass loss in terrestrial and aquatic habitats (Hättenschwiler & Gasser, 2005; Jabiol & Chauvet, 2015; Liu et al., 2020; Santonja et al., 2020; Schädler & Brandl, 2005; Vos et al., 2013). But increased colonization may also be a consequence of the mixture effect, as shredders may benefit from the structured habitat created by recalcitrant species which can also provide shelter against predators or stream flow (Hättenschwiler et al., 2005; Jabiol et al., 2014; Sanpera‐Calbet et al., 2009). Although the mixture had on average 29% more biomass, 16% more abundance and 10% more shredder taxa than the average of the monocultures, mass loss of the mixture was not higher‐than‐expected. Functional redundancy among shredder taxa may be due to dominance by food generalists (Hättenschwiler et al., 2005). Furthermore, the more complex habitat may have also attracted more predators and shredders might have reduced consumption rates as a behavioral response to their presence (Jabiol et al., 2014). However, it is more plausible that there was a similar consumption of both species during decomposition. When given a choice, shredders consume preferentially the highest quality leaves available (Swan & Palmer, 2006). Given that the availability of each species in the mixture changed during decomposition due to the faster breakdown rates of alder, and that N‐concentrations of the two species converged during decomposition (Abelho & Descals, 2019), shredders probably started by consuming alder, but, when mass remaining of alder was already low and the quality of poplar had increased, shifted consumption from alder to poplar.

When the period of terrestrial and aquatic exposures was equal, the process of decomposition of the mixture was enhanced but there was a “dilution” of the effects on each compartment: the effect of the mixture was additive on mass loss, decomposers, and detritivores. However, the partitioning of the contributions of each species within the mixture showed a synergistic effect of the presence of alder on mass loss of poplar. While exposed in the terrestrial habitat, the water buffering effect of the mixture might have increased moisture persistence in the litter layer (Wardle et al., 2003) thus favoring fungal colonization. In fact, although the effect was nonsignificant (but barely), fungal biomass of poplar was elevated in the presence of alder. The conditions in terrestrial habitats are more likely to favor N‐transfer than in streams (Gessner et al., 2010), and it is possible that there was a transference of nitrogen, either passively or actively by fungal hyphae, from alder to poplar (Lummer et al., 2012; Schimel & Hättenschwiler, 2007). But the effect could also have occurred during stream immersion, given that the increase in decomposition rates of nutrient‐poor species by the presence of N‐fixing species has also been shown in aquatic ecosystems (Migliorini & Romero, 2020). However, this species‐specific effect was small and did not translate in a synergistic effect on mass loss of the mixture.

### 4.3 Speculations

As shown here, the effect of mixing litter may be additive for one of the individual compartments (mass loss, decomposers, or detritivores) and even so result in nonadditive effects on the process of decomposition. A measure that includes observations on mass loss, microbial decomposers, and invertebrate detritivores, such as the one used here, provides an integrated assessment of the effect of mixing litter on the process of decomposition.

If the obtained results hold for other streams and other mixtures with contrasting N‐concentrations, and the effect of mixing litter differs between vertical litterfall and lateral inputs, studies on vertical litterfall underestimate the effect of mixing litter on detritivores and overestimate the effect on mass loss and decomposers. Moreover, if applicable elsewhere, the obtained results may also forecast future changes on the effect of mixing litter on decomposition in streams. Climate change predictions are for longer warm seasons associated to a decrease in rainfall over large parts of the subtropics, more intense drought in many regions, and decreased stream flows (Pörtner et al., 2022). In response to hydric stress, deciduous riparian trees are likely to lose their leaves earlier in the season, and a higher proportion of those leaves will fall on the riparian area due to the reduced stream flow. Thus, the prolonged and dry summers are likely to increase the proportion of leaf litter that enters streams through lateral movement and the accumulation of leaves at the riparian area for longer periods of time before they are washed into the water.

## AUTHOR CONTRIBUTIONS

**Manuela Abelho:** Conceptualization (lead); data curation (lead); formal analysis (lead); investigation (lead); methodology (lead); writing – original draft (lead); writing – review and editing (lead). **Enrique Descals:** Investigation (supporting); methodology (supporting); writing – review and editing (supporting).

## FUNDING INFORMATION

This work is carried out at the R&D Unit Centre for Functional Ecology – Science for People and the Planet (CFE), with reference UIDB/04004/2020, financed by FCT/MCTES through national funds (PIDDAC).

## CONFLICT OF INTEREST STATEMENT

The authors have no conflict of interest to declare.

## Data Availability

The data are available in the Dryad Digital Repository (https://doi.org/10.5061/dryad.c59zw3rd9).
